# Increasing Short-Stay Unplanned Hospital Admissions among Children in England; Time Trends Analysis ’97–‘06

**DOI:** 10.1371/journal.pone.0007484

**Published:** 2009-10-15

**Authors:** Sonia Saxena, Alex Bottle, Ruth Gilbert, Mike Sharland

**Affiliations:** 1 Department of Primary Care and Social Medicine, Imperial College London, London, United Kingdom; 2 Centre for Evidence-based Child Health and MRC Centre of Epidemiology for Child Health, UCL-Institute of Child Health, London, United Kingdom; 3 Paediatric Infectious Diseases Unit, St George's Hospital NHS Trust, London, United Kingdom; National Institute for Public Health and the Environment, Netherlands

## Abstract

**Background:**

Timely care by general practitioners in the community keeps children out of hospital and provides better continuity of care. Yet in the UK, access to primary care has diminished since 2004 when changes in general practitioners' contracts enabled them to ‘opt out’ of providing out-of-hours care and since then unplanned pediatric hospital admission rates have escalated, particularly through emergency departments. We hypothesised that any increase in isolated short stay admissions for childhood illness might reflect failure to manage these cases in the community over a 10 year period spanning these changes.

**Methods and Findings:**

We conducted a population based time trends study of major causes of hospital admission in children <10 years using the Hospital Episode Statistics database, which records all admissions to all NHS hospitals in England using ICD10 codes. Outcomes measures were total and isolated short stay unplanned hospital admissions (lasting less than 2 days without readmission within 28 days) from 1997 to 2006. Over the period annual unplanned admission rates in children aged <10 years rose by 22% (from 73.6/1000 to 89.5/1000 child years) with larger increases of 41% in isolated short stay admissions (from 42.7/1000 to 60.2/1000 child years). There was a smaller fall of 12% in admissions with length of stay of >2 days. By 2006, 67.3% of all unplanned admissions were isolated short stays <2 days. The increases in admission rates were greater for common non-infectious than infectious causes of admissions.

**Conclusions:**

Short stay unplanned hospital admission rates in young children in England have increased substantially in recent years and are not accounted for by reductions in length of in-hospital stay. The majority are isolated short stay admissions for minor illness episodes that could be better managed by primary care in the community and may be evidence of a failure of primary care services.

## Introduction

Unplanned hospital admission rates have been rising for several years in many developed countries, [1] particularly in children, many of whom are admitted through emergency departments. [2], [3] Mortality and morbidity from infectious childhood illness have dramatically declined and the likelihood of serious bacterial infection in children is now very low. [4], [5] Despite this, acute conditions such as minor infectious illness remains an important cause for health service use and health expenditure on these conditions has increased.[6], [5] Unplanned admission is expensive, strains resources and can be avoided given timely care by general practitioners (GPs) in the community, which by contrast is cheaper, avoids the hazards of hospital acquired infection and provides better continuity of care than emergency services[7], [8] Where access to primary care is poor, unplanned admission rates are high. [7], [9] In the United States (US), where continuity of care for children is low across primary and secondary health care settings,[10] there have been strong arguments in favour of implementing universal coverage of primary care similar to the United Kingdom (UK) model.[11]

Over 99% of children in the UK are registered with a National Health Service (NHS) general practitioner and most will see their GP at least once each year for mild self-limiting common childhood illnesses, including infection, asthma and minor injury [12] that do not require onward referral to secondary care.[13] Yet in the UK, access to primary care has diminished since 2004 due to restructuring of NHS primary care provision and a change in general practitioners' contracts enabling ‘opt out’ of responsibility for out-of-hours and unplanned care of acute conditions and focusing more on management of chronic conditions in adults.[14] Although consultations on surgery premises have not fallen, the proportion of all GP consultations carried out in patient's homes has halved from 9% to 4%. [15] In 2006/7, 58% of all unplanned admissions to English hospitals in children <19 years old occurred via the emergency route compared with 31% from general practitioners.[3] Since 2004, reductions in working hours of junior hospital doctors[16] have resulted in less-experienced staff in the frontline of emergency care and stringent emergency department waiting time targets have been tightened. [17] This forces staff to make decisions about whether to discharge or admit patients with limited opportunities for review by the same clinician.

We hypothesised that any increase in isolated short stay admissions in young children during the past decade of major reorganisations in primary care in the UK might reflect failure to manage acute childhood illness in the community. This paper examines trends in unplanned hospital admission in children aged less than 10 years over a ten-year period spanning the changes in primary care organisation and describes the pattern of admissions with respect to short hospital stays for minor conditions that could potentially be dealt with in the community. We focused this report on children aged under 10 years because their major causes of admission differ substantially from older children.

## Methods

The Hospital Episodes Statistics (HES) database is a national database operational since 1986, recording all inpatient and day case procedures in NHS (public) hospitals. [18] HES data have been widely used to examine time trends in disease and variations in practice and to make international comparisons (www.hesonline.nhs.uk). We have Section 60 approval from the Patient Information Advisory Group (PIAG) to hold confidential data and analyse them for research purposes. Consent was given on behalf of patients since for national data individual consent is considered unfeasible. We also have approval from St Mary's Local Research Ethics Committee.

The basic unit of the HES dataset is the consultant episode, which covers the period of time during which a patient is under the care of a given consultant and coded using the International Classification of Diseases (ICD) system version 10 to record the main reason for admission as the ‘primary diagnosis’. We used Hospital Episode Statistics data for each year from 1996/7 to 2006/7, to derive unplanned admissions for each calendar year between 1997 and 2006 inclusive. Multiple consultant episodes and hospital transfers were linked to form admissions. We used ICD10 codes for ‘primary diagnosis’ to categorise cause of admission. We included unplanned admissions for children aged 0 to 9 years, creating three age bands: under 1 year, 1–4 years and 5–9 years, reflecting key stages of development in childhood. Mid-year population estimates for age, sex and year were taken from the Office for National Statistics web site to calculate admission rates per 1000 population.[19]

Our aim was to identify short stay unplanned admissions that could potentially have been better dealt with in primary care. We defined a short stay admission as that having a length of stay less than two days. This includes unplanned admissions discharged on the same or the following day. We excluded short stay admissions followed by a readmission within 28 days as these may reflect failure of hospital care.

To rank major causes of admissions, we grouped together ICD10 codes for each admission's primary diagnosis using the 259 AHRQ's CCS (Agency for Healthcare Research and Quality Clinical Classification System) groups.[20] This system aims to classify all ICD codes into 259 clinically meaningful groups and has been used extensively.[21] We ranked the top 5 most common causes for total and short stay admissions in “infectious” and “non-infectious” categories (Appendix S1).

## Results

### Trends in total and short stay unplanned admission rates

Over the ten calendar years 1997 to 2006 there were a total of 1,566,829 unplanned admissions in children aged under 1 year, 2,115,664 in children aged 1–4 years and 1,118,011 in children aged 5–9 years in England.

**Figure 1:**
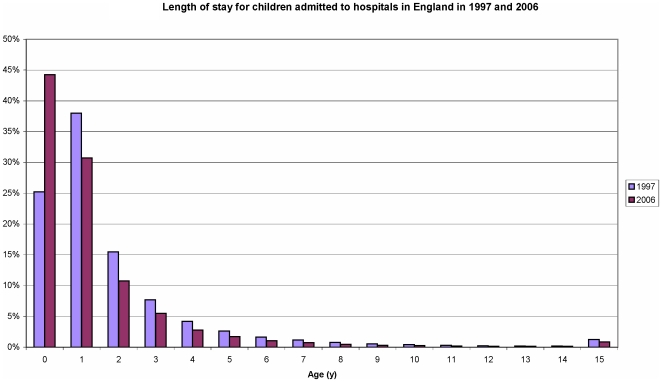
Length of stay for children admitted to hospital aged under 10 years in 1997 and 2006.

The unplanned admission rate for all children under 10 years old increased by 21.6% from 73.6 to 89.5 per 1000 child years from 1997 to 2006. This represents a rise in the incidence rate of 18.4% in children under 1 year, 22.0% in children aged 1–4 years and 15.1% in children aged 5–9 years during that time. However, short stay admission rates increased by 41% from 42.7 to 60.2 per 1000 child years and by 2006 more than two-thirds of admissions in 2006 were short stay (67.3%) compared with 58.1% in 1997 (Table 1). During this period there was a concurrent reduction in the length of stay in all age groups (Figure 1), but the decrease in admissions lasting over 2 days was small from 30.8 to 27.0 per 1000 child years (12% fall). Unplanned readmission rates within 28 days of discharge also rose by 37% from 6.9 to 9.5 per 1000 child years across all age groups (Table 1).

**Table 1:** Overall unplanned admissions and short stay admissions for children <10 years for 1997 and 2006.

Year	Age group (y)	Sex	Number of unplanned admissions	Unplanned admission rate per 1000 population	Short stay admissions* (% of total emergencies)	Rate per 1000 population	Unplanned readmissions within 28 days (% of total emergencies)	Rate per 1000 population
1997	Under 1	Males	89733	285.1	43792 (48.8)	139.2	11581 (12.9)	36.8
		Females	64546	215.7	31734 (49.2)	106.0	7612 (11.8)	25.4
	1–4	Males	115019	90.7	74269 (64.6)	58.6	9178 (8.0)	7.2
		Females	85045	70.7	53901 (63.4)	44.8	6690 (7.9)	5.6
	5–9	Males	65002	39.1	39637 (61.0)	23.9	4992 (7.7)	3.0
		Females	46430	29.3	27175 (58.5)	17.1	3746 (8.1)	2.4
	1997 total		465775	73.6	270508 (58.1)	42.7	43799 (9.4)	6.9
2006	Under 1	Males	102532	333.8	61995 (60.5)	201.8	14137 (13.8)	46.0
		Females	75959	259.5	46349 (61.0)	158.3	9629 (12.7)	32.9
	1–4	Males	131258	109.8	95371 (72.7)	79.8	12684 (9.7)	10.6
		Females	99406	87.1	70612 (71.0)	61.9	9528 (9.6)	8.4
	5–9	Males	66349	44.2	45754 (69.0)	30.5	5654 (8.5)	3.8
		Females	49683	34.6	33532 (67.5)	23.4	4429 (8.9)	3.1
	2006 total		525187	89.5	353613 (67.3)	60.2	56061 (10.7)	

* Short stay unplanned admissions defined as length of stay <2 days and not followed by readmission within 28 days

The increases were particularly marked among children aged less than 1 year and by 2006 the unplanned short stay admission rate had increased by 46.8% from 123 per 1000 to 181 per 1000 child years.

**Figure 2:**
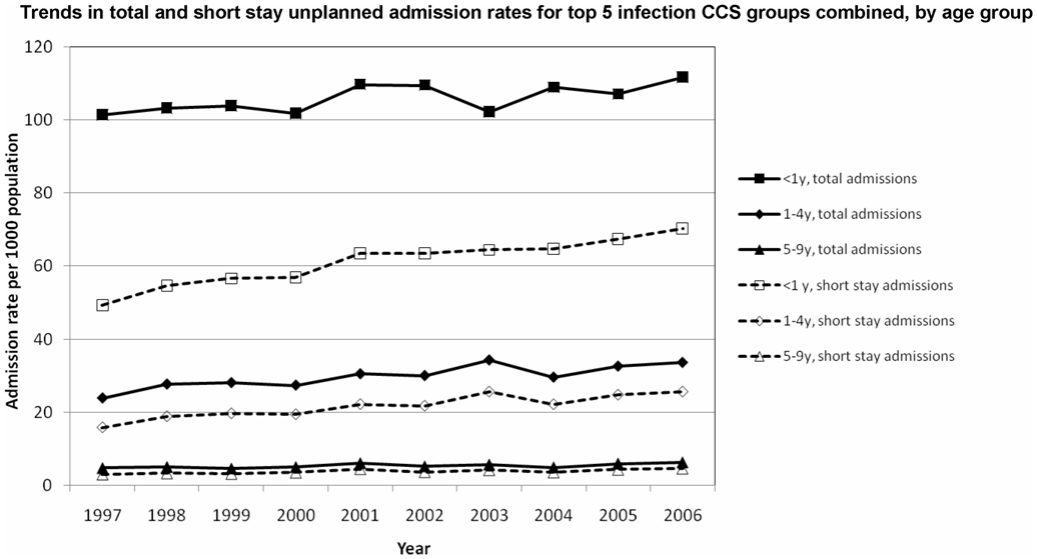
Trends in top 5 infectious causes of unplanned and short stay admissions 1997–2006.

**Figure 3:**
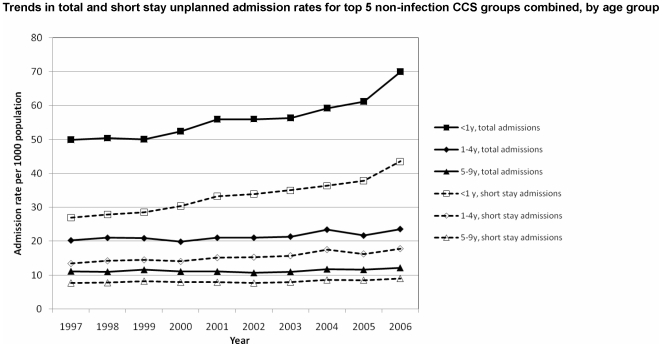
Trends in top 5 non infectious causes of unplanned and short stay admissions 1997–2006.

Figures 2 and 3 show increases in the five commonest non-infectious causes of admissions have increased markedly more than the five commonest infectious causes mainly in children under 5 years and in particular infants aged <1 year.

### Major causes of total and short stay admissions

Appendix S1 shows the top 5 ranked CCS groups of major causes of admission in infectious and non-infectious groups and predominant most common ICD-10 coded cause for admission in each main category. Across each cause of admission category the vast majority of admissions were short stay admissions, with the exception of bronchiolitis in children aged <1 year and pneumonia in children aged 5 to 9 years.

## Discussion

### Main findings

In the period spanning major changes in access to primary care services in the UK (1997 to 2006), unplanned hospital admission rates in young children rose steadily by 22%, with much larger increases of 41% in short stay admissions over the period, particularly among children under 1 year. There was a corresponding smaller fall of 12% in admission rates for length of stay of 2 days or more. By 2006, 67.3% of all unplanned admissions were uncomplicated (i.e. not followed by unplanned readmission) short stays under 2 days. The increases in admission rates were greater for common non-infectious causes than infectious causes of admissions.

### Strengths and limitations

This is to our knowledge the first study to examine specific causes accounting for the burden of short-term childhood illness admitted to English hospitals as emergencies. Its strengths include national coverage over ten years and use of very broad diagnosis categories so minimizing effects of miscoding.[22] However, our study has a number of important limitations. Firstly, the data quality is reliant on the quality of coding and we used primary diagnosis to identify the cause of admission. In some cases the distinction between infectious groups and non-infectious admissions is unclear at aggregate level. More detailed exploration of individual records for each admission, though beyond the scope of this national study, could have revealed more information about comorbidity and reason for admission. Using short stay unplanned admissions as a proxy indicator for identifying minor problems assumes that the admission was inappropriate. However, in some cases a short hospital admission may be justified for severe problems that are resolved quickly, for example a period of observation for head injury. We acknowledge that parents, primary care physicians and hospital physicians have different perceptions of whether children need to be admitted to hospital.[23] Others have used similar coding systems to track potentially preventable hospitalisations for the evaluation of quality of health services. [21] Our findings highlight the need for the NHS in the UK to develop validated indicators to monitor patient pathways though health services and evaluate quality and continuity of care.

### Comparison with previous studies and implications of findings

There are several possible explanations for why unplanned admissions in children have risen over the past 10 years. First, in reducing the length of stay, hospitals may be becoming more efficient at dealing with illness episodes. However, the increase in the proportion of in hospital stays of under 2 days was not accompanied by equivalent decreases in the number of admissions over 2 days. Second, there could be a true increase in morbidity burden in the community. Other studies suggest the proportion of acute illness in children is falling[24] and although many reports are consistent with our findings that a substantial proportion of unplanned admissions still has an underlying infectious cause, [25] rates of infectious illness are declining overall.[26] Since expansion of the primary immunisation schedule in the NHS, the likelihood of serious meningococcal or pneumococcal infection is also falling (www.hpa.org.uk) in line with similar changes in the epidemiology of childhood illness in other countries.[5]

Third, is the rise in emergencies due to a failure in hospitals to adequately deal with childhood illness? Readmissions accounted for less than 10% of total admissions but rates increased by 37% during the ten-year period, which may contribute to some of the overall increase in total unplanned admissions. However, this does not account for the greater increase in short stay admissions where there was no readmission within 28 days. Finally, could the increases in short stay admissions for minor infectious illness be accounted for by changes in the process of care that have impacted on patient pathways? We contend that a range of factors including changes in the accessibility and organization of acute care and health-seeking behaviour by parents have contributed to this rise.

Since the late 1990s, major restructuring of NHS out-of-hours primary care, aimed at reducing demand on general practitioners and emergency departments,[27] shifted overall responsibility for out-of-hours care provision from individual GPs to primary care trusts. There are now many alternatives including telephone-based advice systems (NHS direct), walk-in centres or home visiting served through GP cooperatives or commercial deputizing firms that have potential to reduce immediate workload of GPs. [28] However, few of these offer the continuity of care and opportunities for clinical review afforded by an experienced general practitioner or specialist paediatrician. Most unplanned admissions for children occur out of hours[6] and an increasing proportion of children are admitted to hospital via the A & E route (58% in 2006/7) compared with a falling proportion via a GP (31% in 2006/7).[3] The increase in admissions via A & E might have occurred as a consequence of time pressures on relatively inexperienced junior doctors[29], working shorter shifts[16] and under pressure to achieve A & E waiting time targets with little opportunity for review and continuity of care by an experienced clinician. Thus it is conceivable that junior doctors choose the safer option of admitting children to hospital rather than observing them, or discharging them back to the community. They are then discharged the next morning by the attending consultant. Several recent reports have highlighted inadequacies in training of junior doctors and raised similar concerns about standards of care in NHS hospitals. [30]

Changes in health-seeking behaviour of parents may also have contributed to the rise in admissions. For example, the study period 1997 to 2006 has seen a change in the UK's population age and ethnic mix. Recent migrants are a substantial and diverse group predominantly from other countries within the European Union and one study of emergency department attenders showed they are less likely to use GP services than UK residents.[31] Many parents, particularly those lacking family and social support networks, often find it difficult to distinguish between trivial self-limiting illnesses and more serious conditions, fear the worst when their child is unwell [32] and may require access to support and information from primary medical services to cope.[33] Where access to out-of-hours primary care is limited, parents may seek alternative opinions, duplicating health contacts, straining overstretched accident and emergency services and increasing costs. The impact of rising demands on staff and services from increasing unplanned admissions may even compromise clinical care and put children at risk if junior doctors are under pressure to make rapid decisions on management.[34]

The NHS was born in 1948 out of a long-held ideal of universal access to care from cradle to grave and for years this has been cited as a key factor explaining better health outcomes in the UK compared with those in the US despite spending far less on health care. [11] A central tenet of addressing children's health care needs is providing high quality primary care[35] and the UK's general practitioners have delivered this for many years. Countries looking to adapt the UK model can learn from the rapid reorganisations in the UK that when access to primary care is withdrawn, secondary care may have to take up the strain. Short stay unplanned admissions are expensive, place strain on health services and staff, are disruptive to families and expose children to unnecessary risks. Alternatives to hospital admission include paediatric assessment units,[36] expanded paediatric A&E departments or the more recently introduced general practitioner staffed polyclinics.[37] Any potential solution requires detailed evaluation of clinical and cost-effectiveness by organizations responsible for acute care and managing patient demand [38], [39] to ensure services dealing with acute illness in children do not become an easy target for cost-cutters and do not compromise continuity and quality of care.[30] Admitting ever more children to hospital overnight is probably not the best way forward.

## Supporting Information

Appendix S1 Number of total unplanned and short stay admissions in 2006 for top 5 infection CCS groups and top 5 non-infection CCS groups, by age group Number of total unplanned and short stay admissions in 2006 by age group and five commonest infectious CCS groups and five commonest non-infectious CCS groups with their principal ICD10 codes
(0.16 MB DOC)
