# Supplementary material for: Increasing Short-Stay Unplanned Hospital Admissions among Children in England; Time Trends Analysis ’97–‘06
Source: PLoS One. 2009 Oct 15;4(10):e7484. doi: 10.1371/journal.pone.0007484 (PMC2758998; doi:10.1371/journal.pone.0007484)
Supplement: Appendix S1 — Number of total unplanned and short stay admissions in 2006 for top 5 infection CCS groups and top 5 non-infection CCS groups, by age group Number of total unplanned and short stay admissions in 2006 by age group and five commonest infectious CCS groups and five commonest non-infectious CCS groups with their principal ICD10 codes (0.16 MB DOC) [file pone.0007484.s001.doc]

APPENDIX S1

Number of total unplanned and short stay admissions in 2006 for top 5 infection CCS groups and top 5 non-infection CCS groups, by age group

**Children aged under 1 year**

a) Infection

0

5000

10000

15000

20000

25000

Bronchiolitis

Acute upper

respiratory tract

infections

Viral illness

Intestinal infection

Febrile illness

**Number of emergency admissions**

All admissions

Short stay

b) Non-infection

0

5000

10000

15000

20000

25000

25000

Feeding difficultyy

Breathing difficulty

Haemolytic and

perinatal jaundice

Colic

Vomiting

**Number of emergency admissions**

All admissions

Short stay

**Children aged 1 to 4 years**

a) Infection

0

5000

10000

15000

20000

25000

30000

Acute upper

respiratory tract

infection

Viral illness

Intestinal infection

Acute bronchitis

Acute tonsillitis

**Number of emergency admissions**

All admissions

Short stay

b) Non-infection

0

5000

10000

15000

20000

25000

30000

Convulsions

Breathing difficulty

Asthma

Gastroenteritis

Superficial injury

**Number of emergency admissions**

All admissions

Short stay

**Children aged 5-9 years**

a) Infection

0

2000

4000

6000

8000

10000

12000

Viral illness

Acute upper

respiratory tract

infection

Acute tonsillitis

Intestinal infection

Pneumonia

**Number of emergency admissions**

All admissions

Short stay

b) Non-infection

0

2000

4000

6000

8000

10000

12000

Fracture of upper limb

Asthma

Abdominal pain

Superficial injury

Epilepsy

**Number of emergency admissions**

All admissions

Short stay

Number of total unplanned and short stay admissions in 2006 by age group and five commonest infectious CCS groups and five commonest non-infectious CCS groups with their principal ICD10 codes

| Age group | **CCS group** | **Description** | **Number of unplanned admissions** | **Short stay admissions *** | **% of total unplanned admissions that were short stay** |
| --- | --- | --- | --- | --- | --- |
| Under 1, infectious | 125 | Acute bronchitis (total)  J21 Acute bronchiolitis | 21549  18218 | 9858  8169 | 45.7 |
|  | 126 | Other upper respiratory infections (total)  J06 Acute upper respiratory infections multiple and unspecified sites | 17842  15280 | 13729  11794 | 76.9 |
|  | 7 | Viral infection (total)  B34 Viral infection of unspecified site | 13109  11862 | 8735  7926 | 66.6 |
|  | 135 | Intestinal infection (total)  A08 Viral and other specified intestinal infections | 10606  9121 | 7737  6698 | 72.9 |
|  | 246 | Fever of unknown origin (total)  R50 Fever of unknown origin | 3902  3902 | 2169  2169 | 55.6 |
|  | Other | All other infection CCS groups | 10343 | 4235 | 40.9 |
|  |  | Total | 77351 | 46463 | 60.1 |
| Under 1, non infectious | 224 | Other perinatal conditions (total)  P92 Feeding problems of newborn | 14322  5790 | 7828  3474 | 54.7 |
|  | 134 | Other upper respiratory disease (total)  R06 Abnormalities of breathing | 8313  7812 | 5808  5492 | 69.9 |
|  | 222 | Haemolytic jaundice and perinatal jaundice (total)  P59 Neonatal jaundice from other and unspecified causes | 8156  7844 | 4473  4302 | 54.8 |
|  | 154 | Noninfectious gastroenteritis (total)  K52 Other noninfective gastroenteritis and colitis | 6717  6717 | 4857  4857 | 72.3 |
|  | 250 | Nausea and vomiting (total)  R11 Nausea and vomiting | 4506  4506 | 3167  3167 | 70.3 |
|  | Other | All other non-infection CCS groups | 59126 | 35748 | 60.5 |
|  |  | Total | 101140 | 61881 | 61.2 |
| 1-4, infectious | 126 | Other upper respiratory infections (total)  J06 Acute upper respiratory infections multiple and unspecified sites | 27276  20992 | 22547  17194 | 82.7 |
|  | 7 | Viral infection (total)  B34 Viral infection of unspecified site | 18785  15858 | 14359  12540 | 76.4 |
|  | 135 | Intestinal infection (total)  A08 Viral and other specified intestinal infections | 13801  11856 | 10488  9097 | 76.0 |
|  | 125 | Acute bronchitis (total)  J22 Unspecified acute lower respiratory infection | 10017  8243 | 5860  4887 | 58.5 |
|  | 124 | Acute and chronic tonsillitis (total)  J03 Acute tonsillitis | 8747  8446 | 6828  6613 | 78.1 |
|  | Other | All other infection CCS groups | 23775 | 11936 | 50.2 |
|  |  | Total | 102401 | 72018 | 70.3 |
| 1-4, non infectious | 83 | Epilepsy, convulsions (total)  R56 Convulsions, not elsewhere classified | 14298  11661 | 10129  8862 | 70.8 |
|  | 134 | Other upper respiratory disease (total)  R06 Abnormalities of breathing | 12914  12372 | 10192  9795 | 78.9 |
|  | 128 | Asthma (total)  J45 Asthma | 12841  11683 | 8855  8248 | 69.0 |
|  | 154 | Noninfectious gastroenteritis (total)  K52 Other noninfective gastroenteritis and colitis | 7977  7977 | 5954  5954 | 74.6 |
|  | 239 | Superficial injury, contusion (total)  S09 Other and unspecified injuries of head | 7110  4289 | 6441  3939 | 90.6 |
|  | Other | All other non-infection CCS groups | 73123 | 52394 | 71.7 |
|  |  | Total | 128263 | 93965 | 73.3 |
| 5-9, infectious | 7 | Viral infection (total)  B34 Viral infection of unspecified site | 5099  4284 | 3951  3479 | 77.5 |
|  | 126 | Other upper respiratory infections (total)  J06 Acute upper respiratory infections multiple and unspecified sites | 4818  3249 | 3919  2627 | 81.3 |
|  | 124 | Acute and chronic tonsillitis (total)  J03 Acute tonsillitis | 3033  2842 | 2328  2216 | 76.8 |
|  | 135 | Intestinal infection (total)  A08 Viral and other specified intestinal infections | 3008  2384 | 2315  1915 | 77.0 |
|  | 122 | Pneumonia (except that caused by tuberculosis or STD) (total)  J18 Pneumonia, organism unspecified | 2756  2492 | 979  879 | 35.5 |
|  | Other | All other C infection CS groups | 9245 | 4873 | 52.7 |
|  |  | Total | 27959 | 18365 | 65.7 |
| 5-9, non infectious | 229 | Fracture of upper limb (total)  S52 Fracture of forearm | 10507  7355 | 7726  5560 | 73.5 |
|  | 128 | Asthma (total)  J45 Asthma | 8730  7833 | 5643  5203 | 64.6 |
|  | 251 | Abdominal pain (total)  R10 Abdominal and pelvic pain | 7176  7176 | 5498  5498 | 76.6 |
|  | 239 | Superficial injury, contusion (total)  S09 Other and unspecified injuries of head | 4729  2726 | 4332  2552 | 91.6 |
|  | 83 | Epilepsy, convulsions (total)  R56 Convulsions, not elsewhere classified | 4419  2203 | 3152  1758 | 71.3 |
|  | Other | All other non-infection CCS groups | 52512 | 34570 | 65.8 |
|  |  | Total | 88073 | 60921 | 69.2 |
